# Osteochondral impaction of the posterior acetabular surface without cortical fracture of any wall or column: an undescribed pattern of acetabular injury

**DOI:** 10.1007/s10195-011-0139-x

**Published:** 2011-05-17

**Authors:** R. Pascarella, V. Digennaro, G. Grandi

**Affiliations:** Orthopedics and Traumatology Department, Maggiore Hospital, Largo B.Nigrisoli 2, 40133 Bologna, Italy

**Keywords:** Acetabular fracture, Quadrilateral lamina, CT scan, Kocher–Langebeck

## Abstract

Surgical treatment of a unusual acetabular fracture is described. This fracture was characterized by impaction and breaking down of the posterior articular surface and comminution of *lamina quadrilatera* lower portion, without cortical fracture of both columns. The fracture was treated surgically through the Kocher–Langenbeck approach. A small hole was created in the acetabulum posterior wall, the impacted fragment was reduced, and the bone defect was filled with autologous bone from the greater trochanter. A plate was shaped in order to fix both bone graft and fractured fragment.

## Introduction

Acetabular fracture types are usually determined by two classifications: Arbeitsgemeinschaft fuer Osteosynthesefragen - Association for the Study of Internal Fixation (AO/ASIF) and Leotournel and Judet (L-J). According to the L-J classification [4], acetabular fractures are divided into two types: simple and combined. The five elemental fractures are: (a) posterior wall, (b) posterior column, (c) anterior wall, (d) anterior column, and (e) transverse. The five combined fractures are: (a) posterior column and wall, (b) transverse and posterior wall, (c) anterior column and posterior hemitransverse, (d) T-type, and (e) both columns. The AO classification [9] describes three types of fracture: (a) type A, partial articular, involving only one of the two columns, which includes three subgroups: A1, posterior wall fracture, A2, posterior column, and A3, anterior column or wall; (b) type B, partial articular, involving the transverse component, which includes B1, pure transverse; B2, T-shaped; and B3, anterior column and posterior hemitransverse; and (c) type C, which are complete articular fractures and involve both columns, including C1, high variety, extending to the iliac crest; C2, low variety, extending to the anterior border of the ilium; and C3, extension into the sacroiliac joint. However, transitional types of acetabular fracture might occur, which are difficult to classify but can often be matched to well-described fractures. The acetabular fracture reported in this case is not described in the AO/ASIF or L-J classification and cannot be matched to a transitional type fracture.

## Case report

A 46-year-old man in good physical health was admitted to another hospital following a car accident. Some days after the trauma, he was sent to our surgery center. The patient presented an impacted fracture of the posteroinferior articular surface of the acetabulum with comminution of the quadrilateral lamina. Both anterior and posterior columns were intact. Surgical treatment was performed using the Kocher–Langenbeck approach, and the patient was placed in the prone position. The posterior column of the acetabulum did not show cortical interruption, and so a small hole was made using the scalpel to lift up the posterior wall. When the impacted fragment was retrieved, an autologous bone graft from the greater trochanter was used to fill the bone defect. The osteochondral fragment was repositioned. After filling the bone, the fragment was unstable due to quadrilateral lamina comminution and ischiatic bone fracture. A four-hole plate was shaped like a bracket to hold the osteochondral fragment in place because it tended to fall out. Then the plate was locked with a screw in the ischiatic bone. In addition, the fragment of posterior wall made by the osteotomy was replaced and stabilized with a six-hole plate. Postoperatively, a computed tomography (CT) scan was performed to check the position of the impacted fragment.

Three years after surgery, the patient had a normal hip range of motion without pain. No limitation in activities of daily living was reported, and now the patient plays American football. The patient gave his consent to publish this case report.

## Discussion

The acetabular fracture described in this case report cannot be placed in the AO/ASIF or L-J classifications or be matched to transitional types. In this case, both columns were intact, but there was a deep impaction with dislocation of the posteroinferior articular portion and comminution of quadrilateral lamina and ischiatic bone fracture. A posterior wall fracture is characterized by the presence of an impacted articular fragment. In young men, this fracture is often combined with hip dislocation, thus provoking trabecular bone compression and osteochondral fragment impaction [7]. In patients over 50 years of age, especially in women, osteochondral impaction also occurs without hip dislocation due to reduced bone strength, as in osteoporotic bone or fractures in the elderly [2, 3, 6, 8].

**Fig. 1:**
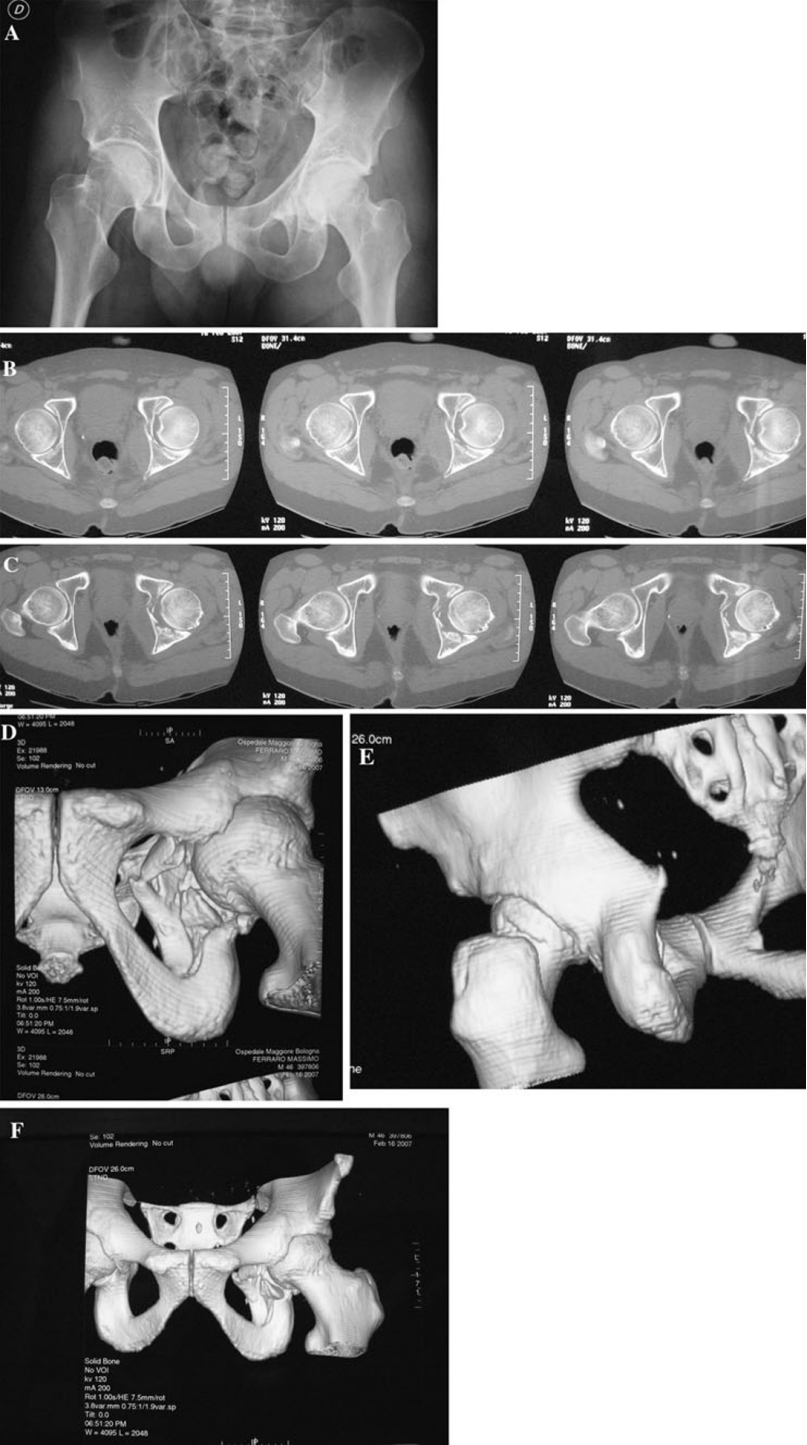
**a** X-ray of the pelvis showing fracture of upper part of the ischium in the lateral part of the obturator foramen. **b**, **c** Computed tomography (CT) showing the impacted fragment of the articular surface and comminution of the*lamina quadrilatera*. **d**–**f** 3D CT highlighting the well-impacted articular fragment and involvement of the*lamina quadrilatera*. The anterior and posterior columns are intact

**Fig. 2:**
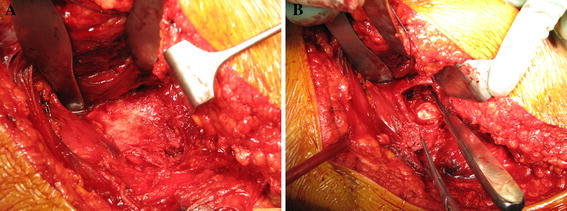
**a**, **b** Intraoperative image of the intact posterior column. After osteotomy of the posterior wall, the impacted fragment was reduced

**Fig. 3:**
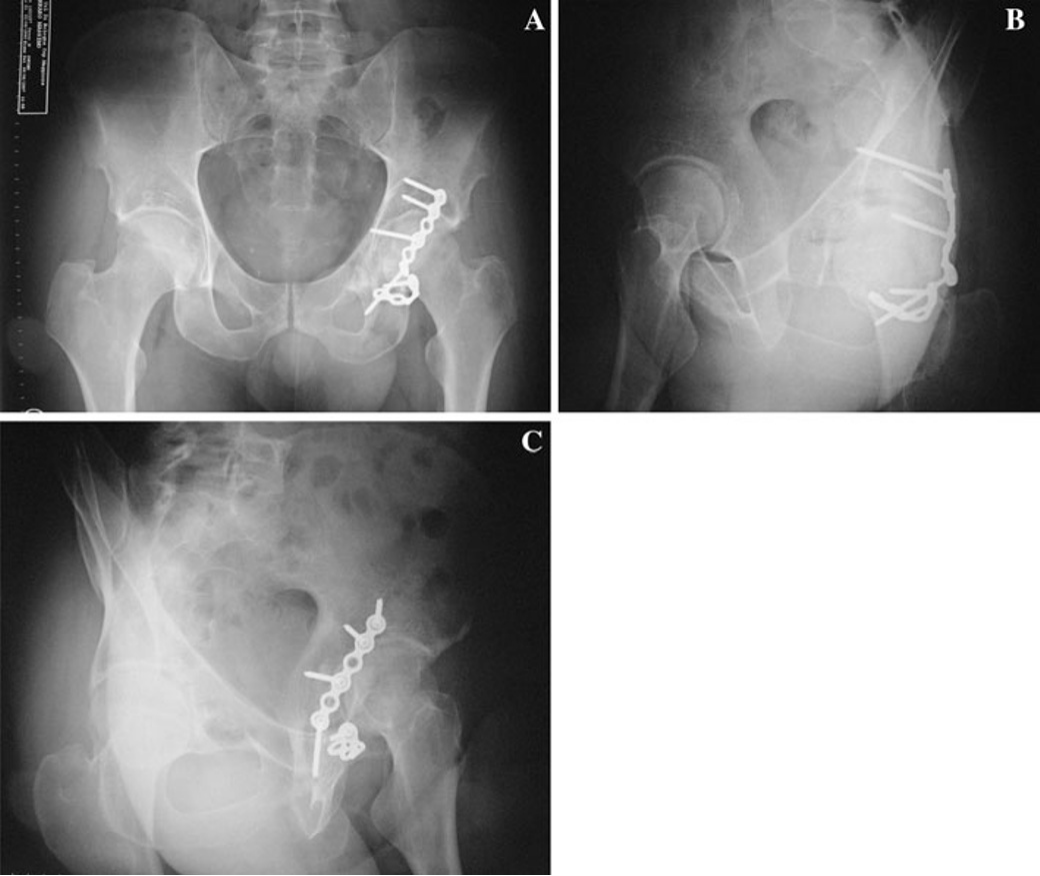
**a**–**c** Postoperative X-ray. One plate is on the posterior column to maintain reduction of the posterior wall. The second plate is under the ischium to maintain reduction of the intra-articular fragment and a fragment of the*lamina quadrilatera*

**Fig. 4:**
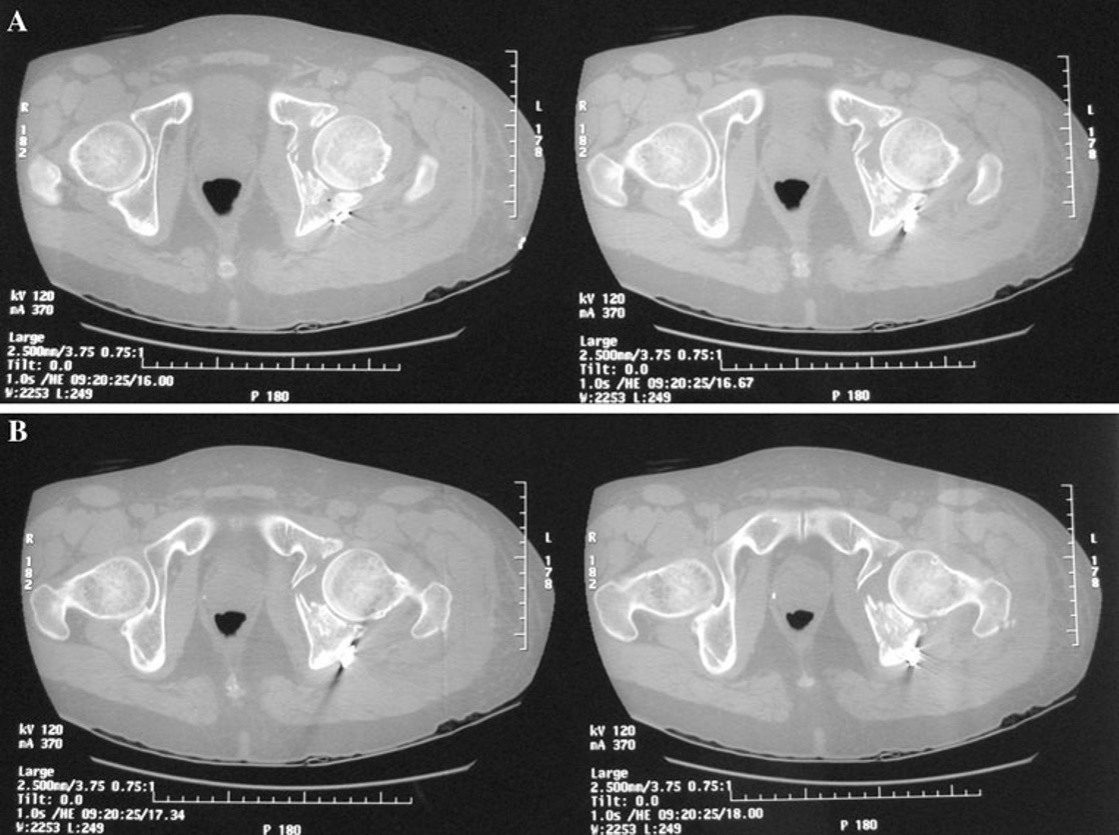
Postoperative computed tomography highlighting reduction of the intra-articular impacted fragment

**Fig. 5:**
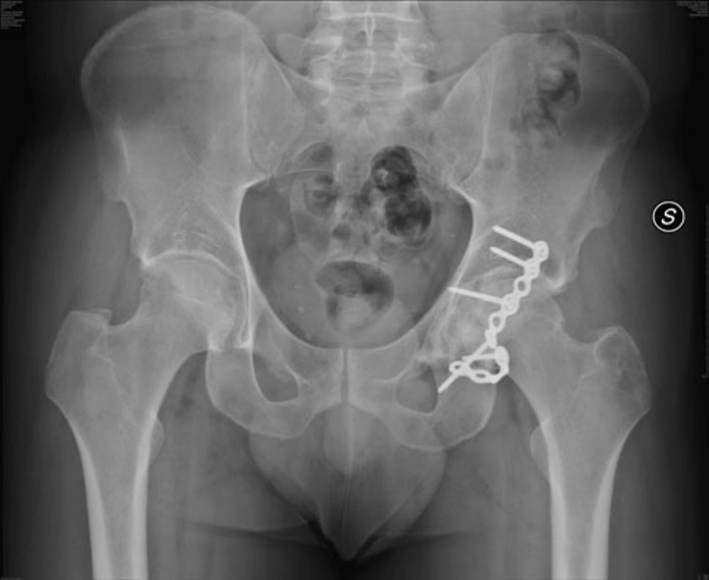
X-ray after 3 years showing a good clinical result

Quadrilateral lamina fracture is often combined with anterior column/wall fracture and fracture of both acetabular columns. In this case, both columns were completely intact, and so it was not referable to known acetabular fracture types. After the uniqueness of this fracture had been verified, the choice of treatment had to be made. First was whether to perform conservative or surgical treatment. Impaction of the posteroinferior articular surface might lead to rapid osteochondral degeneration with secondary hip arthritis. To help determine whether it was better to perform surgical treatment and reduce the impacted fragment, a CT scan of the acetabulum was performed and carefully analyzed and showed a large impaction of articular fragment. An articular fracture was detected in eight CT-scan slides involving >2 cm of the articular surface, as each CT-scan slide measures 3 mm. Therefore, the decision to perform a surgical reduction of the fracture was reached (Figs. 1, 2, 3, 4, 5).

With regard to the surgical approach, the ilioinguinal approach combined with the Stoppa approach [1, 5] enables a quadrilateral lamina fracture to be detected, but in this case, there was not enough space to see the impacted fragment, reduce, and stabilize it with a plate. Therefore, the posterior approach (Kocher–Langenbeck) was used. Three years after surgery, the patient had a normal hip range of motion, no pain or limitation in activities of daily living, and now plays American football. Managing a unique kind of acetabular fracture is reported in this article. Due to its rarity, it was extremely hard to decide whether to treat it surgically and, if so, by which approach. We concluded that the surgical technique chosen allowed adequate reduction of the articular fragment, good stability of the fracture, and an excellent clinical outcome 3 years after the operation.
